# Novel insights into the aetiology and pathophysiology of increased airway inflammation during COPD exacerbations

**DOI:** 10.1186/1465-9921-7-80

**Published:** 2006-05-22

**Authors:** Maria Tsoumakidou, Nikolaos M Siafakas

**Affiliations:** 1 Department of Thoracic Medicine, Medical School, University of Crete, Greece; 2 Lung Pathology Unit, Department of Gene Therapy, National Heart & Lung Institute, Imperial College, London, UK

## Abstract

Airway inflammation increases during acute exacerbations of COPD. Extrinsic factors, such as airway infections, increased air pollution, and intrinsic factors, such as increased oxidative stress and altered immunity may contribute to this increase. The evidence for this and the potential mechanisms by which various aetiological agents increase inflammation during COPD exacerbations is reviewed. The pathophysiologic consequences of increased airway inflammation during COPD exacerbations are also discussed. This review aims to establish a cause and effect relationship between etiological factors of increased airway inflammation and COPD exacerbations based on recently published data. Although it can be speculated that reducing inflammation may prevent and/or treat COPD exacerbations, the existing anti-inflammatory treatments are modestly effective.

## Background

Exacerbations are a cardinal feature of the natural history of moderate and severe Chronic Obstructive Pulmonary Disease (COPD)[1]. Patients with frequent exacerbations have significantly lower quality of life and increased morbidity and mortality rates [1-4]. Although the effect of COPD exacerbations on lung function has been questioned in the past, recent evidence suggests that exacerbations accelerate long-term decline in lung function, specifically in smokers [5-7]. The mechanism of this acceleration remains largely unknown.

Treatment decisions for COPD patients are frequently made according to exacerbation rates. In the ATS/ERS guidelines it is stated that patients with frequent exacerbations should be initiated a trial of inhaled steroids[8]. Systemic steroids should be administered during acute COPD exacerbations, both in the inpatient and outpatient setting. These treatment strategies indicate the central role of inflammation in the pathogenesis of exacerbations.

Although evidence for increased inflammation on COPD exacerbations has been reviewed previously, there has been little focus on why inflammation increases and which the consequences of this increase are[9,10]. Investigating further these important questions may help establish a cause and effect relationship between inflammation and COPD exacerbations. The primary aim of this review is to summarize emerging explanations for *why *inflammation is increased during COPD exacerbations. For practical purposes an attempt is made to categorize aetiological factors of increased inflammation into extrinsic or intrinsic, as it can be seen in table 1. A secondary aim is to try to explain *how *increased inflammation is associated with the pathophysiology of COPD exacerbations.

**Table 1:** Aetiology of increased airway inflammation during COPD exacerbations

**EXTRINSIC FACTORS**	**INTRINSIC FACTORS**
**1. Acute airway infections** a. Bacterial *H. Influenzae* *S. pneumoniae* *M. Cattarhalis* b. Viral *Rhinoviruses* *Picornaviruses* *RSV* *Influenza A, B* *Coronaviruses* c. Atypical *C. Pneumonia* **2. Air pollution**	**1. Oxidative stress** **2. Altered immunity** *Tc1/Tc2 imbalance* **3. Increased inflammation on stable state** *Bronchiectasis* *Bacterial Colonization*

## Aetiology of increased inflammation on exacerbations

### Extrinsic factors

#### 1. Airway infections

##### 1a Bacterial infections

Bacterial infections are generally considered to be the most common causes of COPD exacerbations. It is estimated that more than 40% of all exacerbations are of bacterial origin[9,11,12]. Accordingly, antibiotics should be administered in inpatients and outpatients with acute COPD exacerbation and changes in sputum characteristics suggestive of bacterial infection[8].

The most common bacteria connected to COPD exacerbations are non-typable H. Influenzae, S. Pneumoniae, and M. Cattarhalis[9,11,12]. The same bacteria often colonize the nasal mucosa and pharynx of healthy individuals, but in smokers and in patients with COPD impaired mucocilliary clearance and innate immunity allow these pathogens to colonize the lower airways[13]. COPD exacerbations may be triggered by the acquisition of a new bacterial species or by an increase in the absolute number of the same bacteria that colonize the airways or by the acquisition of a different strain from the same bacterial species [14-16].

Airway bacteria initiate airway inflammation through several interconnecting mechanisms. The surface of bacteria allows the complement system to be activated through the alternative pathway, while specific surface molecules of the bacteria, called Pathogen-Associated Molecular Patterns (PAMPs), bind to pattern recognition receptors on a variety of leukocytes and initiate signalling pathways that lead to the activation of NF-κB and production of proinflammatory cytokines[17]. Once activated, innate immunity can trigger both cell-mediated and antibody-mediated adaptive immune responses. This cascade of events leads to increased blood flow to tissue, increased temperature, redness and swelling which characterize inflammation.

A significant number of studies in stable COPD patients suggest that airway bacterial infections are associated with increased airway inflammation(18–21). Finding a relationship between bacteria and inflammation on stable COPD adds weight to the argument that bacteria may play a causative role in airway inflammation during COPD exacerbations.

Soler et al used protected specimen brush and bronchoalveolar lavage sampling to determine inflammatory cell counts, levels of cytokines concentrations and microbial patterns in stable COPD patients and found that increased neutrophils and tumour necrosis factor-alpha (TNF-alpha) levels may be related to bronchial colonization[18]. Increased TNF-alpha, as well as myeloperoxidase (MPO) and interleukin-8 (IL-8) levels have been specifically related with H. Influenzae infection, as shown by Bresser et al[19]. However, in that study all mediators were measured in frozen sputum and MPO and IL-8 levels were only retrospectively compared to non-infected patients. In fresh sputum samples from COPD patients R. Stockley and his group demonstrated that MPO, neutrophil elastase (NE) activity, IL-8 and LTB4 levels are positively related to sputum bacterial load[20]. Moreover, the type of organism affected sputum MPO levels and NE activity; MPO levels were relatively increased in the presence of Ps. Aeruginosa compared to H. Influenzae and to M. Catarrhalis. There have been also reports for decreased secretory leukocyte protease inhibitor (SLPI) in sputum samples from COPD patients colonised with bacteria[20,21]. Upper airways inflammation in COPD is also increased when there is bacterial colonization[22]. All these results taken together suggest that bacteria are actively involved in the mechanisms of increased inflammation in stable COPD. It would be logical to assume a similar association for COPD exacerbations.

A large prospective longitudinal study by Sethi et al addressed the hypothesis that patients with bacterial-positive exacerbations show increased inflammation compared to bacterial-negative exacerbations[23]. Among H. Influenzae, H. Parainfluenzae and M. Catarrhalis positive exacerbations, H. Influenzae and M. Catarrhalis demonstrated higher sputum TNF-alpha and NE levels compared to bacterial-negative exacerbations. Moreover, increased NE levels above a certain level could distinguish bacterial from non-bacterial exacerbations with 71% sensitivity and 84% specificity. Others have failed to report any difference in sputum NE levels and other fluid-phase mediators between patients with H. Influenzae exacerbations or bacterial-negative exacerbations[24]. This discrepancy may be due to differences in sputum induction time or in sputum processing or in the assays used for the detection of fluid-phase mediators. Another intriguing hypothesis is that different strains of the same pathogens may induce different levels of inflammation and subjects taking part in different studies might have been infected by different strains of H. Influenzae. It was recently shown that H. Influenzae strains isolated from COPD patients during exacerbation induce more inflammation than strains of the same pathogen isolated from colonizers[16]. The close association between airway infections and increased inflammation during COPD exacerbations has been further confirmed by a report of increased systemic inflammation in infected patients during exacerbation[25].

Consistent with these observations, airway inflammation can be decreased with treatment of the infection. Early evidence came from a relatively small study, which showed that neutrophilic mediators' levels may decrease after treatment of bacterial exacerbations[26]. Gompertz et al confirmed that there are significant decreases in neutrophilic inflammatory mediators after treatment of purulent exacerbations[27]. Most importantly, White et al studied patients with bacterial exacerbations and demonstrated a significant fall in sputum leukotriene B4 (LTB4) levels and an increase in SLPI levels in patients in whom bacteria were eradicated, but not in those in whom bacteria persisted on stable state[24]. Moreover, MPO and LTB4 levels were significantly lower and SLPI levels significantly higher in patients with treated compared to patients with untreated bacterial infections on stable state.

In conclusion, three major findings support the hypothesis that bacterial infections are actively implicated in the mechanisms of increased airway inflammation during COPD exacerbations: 1) bacterial infections increase airway inflammation in colonized stable COPD patients 2) bacterial-positive exacerbations show increased inflammation (particularly of neutrophilic type) compared to bacterial-negative exacerbations and 3) eradication of bacteria after a bacterial exacerbation is accompanied by a significant decrease in airway inflammation. A summary of these findings is presented in table 2.

**Table 2:** Studies showing an association between bacterial infections and airway inflammation in stable COPD and on exacerbations

**Year**	**Author**	**Main finding**
*1999*	*Soler*	Increased NEU, TNF-a in colonized pts
*2000*	*Bresser*	Increased TNF-a, MPO, IL-8 in H. Influenzae colonized pts
*2000*	*Hill*	Positive relation between MPO, NE, IL8, LTB4 and bacterial load in stable state. Increased MPO and decreased SLPI in Ps. Aeroginosa colonized pts
*2000*	*Sethi*	Increased TNFa, NE in H. Influenae and M. Catarrhalis exacerbations
*2000*	*Crooks*	Decrease in MPO, IL8, LTB4, after treatment of bacterial exacerbations
*2001*	*Gombertz*	Decrease in LTB4 after treatment of purulent exacerbations
*2002*	*Patel*	Positive relation between IL8 and bacterial load. Decreased SLPI in colonized pts
*2003*	*White*	Decrease in LTB4, increase in SLPI after bacteria eradication on exacerbations

##### 1b Viral infections

Respiratory viruses are important triggers of COPD exacerbations. Initial studies using serology and cell cultures for detecting viral infections suggested that 30% of COPD exacerbations are related to viral infections [28-30]. Later studies using the more sensitive method of reverse transcriptase polymerase chain reaction showed that 40%–50% of COPD exacerbations may be secondary to viral infection[31,32]. Rhinoviruses, picornaviruses, respiratory syncytial virus, influenza A and B and coronaviruses are more frequently detected[31,32].

Possible mechanisms of viral-induced inflammation have been described. The airway epithelial cell is the principal host cell for most respiratory viruses[33]. Viral replication in the epithelial cell triggers intracellular signalling pathways, including activation of NFκB, which leads to increases in the secretion of multiple cytokines and recruitment of multiple leukocytes to the airways[33]. Antigen presenting cells are of particular importance, because binding of viruses to these cells induces innate and adaptive immune responses and T lymphocyte activation[34].

There is convincing data that viruses induce inflammation in asthma[35]. In animal models of emphysema, latent adenoviral infection amplifies the emphysematous destruction and increases the inflammatory response[36]. In stable COPD latent adenoviral infection has been associated with severe emphysema and increased inflammation[37]. The group of J Wedzicha showed that viral infections might be implicated in the mechanisms of increased airway and possibly systemic inflammation during COPD exacerbations. Plasma IL-6 and fibrinogen levels were higher during viral than non-viral exacerbations, although the difference just failed to reach statistical significance[31]. However, in that study viruses were detected in nasal samples and it has been shown that in COPD patients respiratory viruses are detected more frequently in induced sputum than in nasal lavage[32,38]. When rhinovirus infection was detected in induced sputum samples a significant correlation was demonstrated between rhinovirus infection and increased sputum IL-6 levels on COPD exacerbations[38].

Further to these observations, two recent studies showed that viral airway infections during COPD exacerbations are related to airway eosinophilia[39,40]. This new finding may be of particular clinical importance as airway eosinophilia could be used as an indicator of viral infection during an exacerbation. The important role of eosinophils in the pathogenesis of COPD exacerbations is further supported by studies in bronchial biopsies and sputum samples, which show increased eosinophil numbers and eosinophil mediators in COPD patients during exacerbations [41-46]. Eosinophils may be actively involved in the pathogenesis of viral-induced COPD exacerbations through the release of destructive enzymes, reactive oxygen species and inflammatory mediators.

##### 1c Atypical bacteria

Atypical pathogens with potential importance in acute exacerbations include M. Pneumoniae, C. Pneumoniae and Legionella spp. Considerable confusion exists in the literature regarding the significance of these potential pathogens in acute exacerbations of COPD[47]. This is partly due to differences in the techniques used to detect the presence of atypical infections. When a fourfold increase in antibody titter or a positive culture or RT-PCR is used, M. Pneumoniae and Legionella are rare and C. Pneumoniae infection may be involved in up to 9% of COPD exacerbations[30,48-50]. Moreover, chronic colonization with C. Pneumoniae may be associated with a higher rate of COPD exacerbations[51]. C. Pneumonia infection can amplify inflammation in the airways of COPD patients by stimulating the production and expression of cytokines, chemokines and adhesion molecules[52]. However, clear evidence showing a direct relationship between increased inflammation and C. Pneumoniae infection during COPD exacerbations is yet lacking[50].

#### 2. Increased air pollution

Epidemiologists have linked ambient particulate air pollution (PM) exposure with exacerbations of pre-existing pulmonary diseases, such as COPD[53]. PM-mediated enhancement of airway inflammation is a central pathogenetic mechanism by which PM exposure leads to exacerbation of inflammatory pulmonary diseases [54-56]. It has been suggested that PM exposure induces lung inflammation by an increase in Reactive Oxygen Species[57,58]. To the best of our knowledge there are no *in vivo *studies on the effect of PM exposure on airway inflammation in COPD patients. There is also lack of information on airway inflammation during PM exposure induced COPD exacerbations, which may be due to difficulties in defining such exacerbations.

### Intrinsic factors

#### 1. Increased oxidative stress

An imbalance between oxidants and antioxidants may be involved in the development of COPD exacerbations. Almost a decade ago Rahman et al showed that plasma Trolox equivalent antioxidant capacity is decreased in patients presenting with acute exacerbation of COPD[59]. Recent reports suggest that 8-isoprostane levels are increased in exhaled breath condensate of COPD patients during exacerbations, while levels of the antioxidant enzyme glutathione(GSH) in bronchoalveolar lavage fluid are decreased[60,61]. Oxidative stress may be closely associated to increased inflammation during exacerbations[60,62].

Oxidant stimuli induce cellular expression of inducible nitric oxide synthase and heme-oxygenase-1(HO-1) and increase nitrotyrosine formation. We have shown that there is increased HO-1 expression and nitrotyrosine formation in the airways of COPD patients during severe exacerbations relatively to stable state and that this is accompanied by an increase in indices of neutrophilic inflammation, i.e. neutrophil numbers, MPO and IL-8 levels[62]. Evidence for a close association between oxidative stress, airway neutrophilia and increased IL-8 during severe COPD exacerbations is also supported by Drost et al[60]. A certain limitation in the study by Drost et al is the fact that different patients were examined on exacerbation and on stable state.

Oxidative stress induces the transcription of various inflammatory factors, such as nuclear factor-kappaB (NF-κB) and activator protein-1 (AP-1)[63]. It has been shown that NF-κB DNA binding in sputum inflammatory cells is increased during COPD exacerbations[64]. Other investigators have reported increased nuclear localisation of p65, which is a signal of NF-κB activation, in sputum macrophages during COPD exacerbations[65]. NF-κB is important for transcription of IL-8 gene and oxidative stress may induce or amplify airway neutrophilia by inducing the transcription of IL-8 gene[66]. In support of this hypothesis both we and Drost et al have found increased IL-8 levels and neutrophil numbers associated with increased oxidative stress on severe COPD exacerbations[60,62].

Activated neutrophils and other inflammatory cells can in turn release reactive oxygen species and increase airway oxidative stress. Because oxidative stress can induce inflammation and vice versa, it is not clear which of the two, oxidative stress or inflammation, is primarily involved in the mechanisms of COPD exacerbations-[the chicken and egg problem].

#### 2. Altered immunity

Alterations in innate and adaptive immunity are implicated in COPD pathogenesis[67]. Evidence favouring participation of the adaptive immune response in COPD includes the several reports of increased numbers of T-lymphocytes, specifically CD8+ve T-cells with a "type 1"profile [68-71]. It is still unknown whether these alterations are triggered by cigarette smoking, viral infections, or there is a genetic predisposal. We have recently shown that CD8+ve T lymphocytes may mediate their destructive effects in COPD through increased perforin expression and cytotoxic activity[72]. It would be reasonable to assume that lymphocytes may be implicated in the mechanisms of increased inflammation during COPD exacerbations.

According to our observations changes in lymphocyte subpopulations occur during severe COPD exacerbations[73]. In specific, there is a further increase in CD8+ve T cells and this is rather associated with increased CD8+ve type 2 cells compared to type 1. This finding suggests that Tc1 and Tc2 responses may fluctuate in relation to the different phase (exacerbation versus stable state) of the disease in the same patient. Similar observations have been made in other inflammatory diseases[74]. A relative increase of Tc2 versus Tc1 cells may result in impaired immunity, increased susceptibility to viral infections and increased inflammation [75-77].

Although Saetta et al also found increased T cell numbers in endobronchial biopsies from chronic bronchitis patients on mild exacerbations, no difference was detected in CD4 or CD8+ve cell numbers[45]. This discrepancy may be attributed to the fact that we examined sputum samples (not biopsies) from COPD patients (not chronic bronchitis) on severe exacerbation (not mild exacerbation). Moreover, Saetta et al compared different patients on exacerbation and on stable state, which could be a limitation in their study. However, finding increased T cell numbers still adds evidence to the argument that immune responses may be involved in the mechanisms of increased airway inflammation during COPD exacerbations.

#### 3. Increased baseline levels of inflammation

It would be logical to assume that small increases in airway inflammation in patients with already increased baseline levels of inflammation can easily trigger a COPD exacerbation. This presumes the existence of an inflammatory threshold, above which exacerbation occurs. In relation to this hypothesis, Bhowmik et al showed that COPD patients with frequent exacerbations (≥ 3 episodes/year) have increased baseline sputum IL-6 and IL-8 levels[78]. However, it was not examined whether this was related to bacterial colonization, as bacterial colonization can increase airway inflammation and exacerbation rates. The same group also showed faster rises over time in plasma fibrinogen and sputum IL-6 in patients with frequent exacerbations[79].

On the contrary, Gombertz et al did not detect any difference in neutrophilic mediators (including IL-8) between frequent (≥ 3 episodes/year) and infrequent exacerbators[80]. Interestingly, when patients with bronchiectasis were excluded from the analysis, SLPI was found to be lower in COPD patients with frequent exacerbations, which suggests that COPD patients with bronchiectasis may represent a distinct group. Fujimoto et al also report no difference in baseline IL-8 and markers of eosinophilic inflammation between stable and unstable COPD patients[42]. However, results between this and other studies are not comparable due to lower exacerbation rates (mean 1 episode/year) in the unstable COPD group in this study.

In conclusion, data relating baseline airway inflammation to exacerbation frequency are rather controversial. Part of the existing confusion may be due to significant heterogeneity among COPD patients and to the existence of several factors, like bacterial colonization and bronchiectasis that may increase airway inflammation. In particular, COPD patients with bronchiectasis may represent a distinct group characterized by higher rates of bacterial colonization, increased baseline levels of airway inflammation and longer symptom recovery times at exacerbation[81].

### Inflammation and pathophysiology of exacerbations

Episodes of COPD exacerbations are characterized by an acute increase in a patient's baseline dyspnoea, cough and/or sputum production[8]. Severe exacerbations are also associated with worsening of pulmonary gas exchange that may lead to hypoxemia with or without hypercapnia[82]. In order to support a role for airway inflammation in COPD exacerbations the mechanisms which are induced by airway inflammation need to be related to the symptoms and pathophysiology of exacerbations.

Increased airway inflammation induces many pathologic changes on the airways. Accumulation of inflammatory cells in the airway mucosa by itself causes airway wall thickening. Inflammatory cells can release potentially harmful mediators, such as proteases and reactive oxygen species[83]. Neutrophil and eosinophil products, like MPO, NE and eosinophilic cationic protein (ECP), have been found increased on exacerbations, and can cause inflammatory damage and increased permeability of the bronchial mucosa, resulting in airway oedema and protein exudation[20,42,62,84]. Inflammatory mediators, like ECP, can also induce bronchoconstriction by increasing achetinocholine release from parasynmpathetic nerves, while others, like NE, increase mucus secretion[85,86]. Furthermore, mediators of inflammation enhance coughing[87]. Potentially harmful mediators may be released not only by inflammatory cells but also by resident cells. For example, endothelin (ET)-1, which has been found increased on exacerbations, can be released by epithelial cells and stimulates mucus secretion and bronchial hyperesponsiveness[50]. The cascade of the above events leads to significant airway narrowing and increased airway secretions, while the patient suffers from increased cough, sputum, and/or increased dyspnea. The mechanisms of dyspnea are not entirely understood yet, but may be also associated with excessive airway narrowing and dynamic hyperinflation due to increased inflammation [88-90].

**Figure 1:**
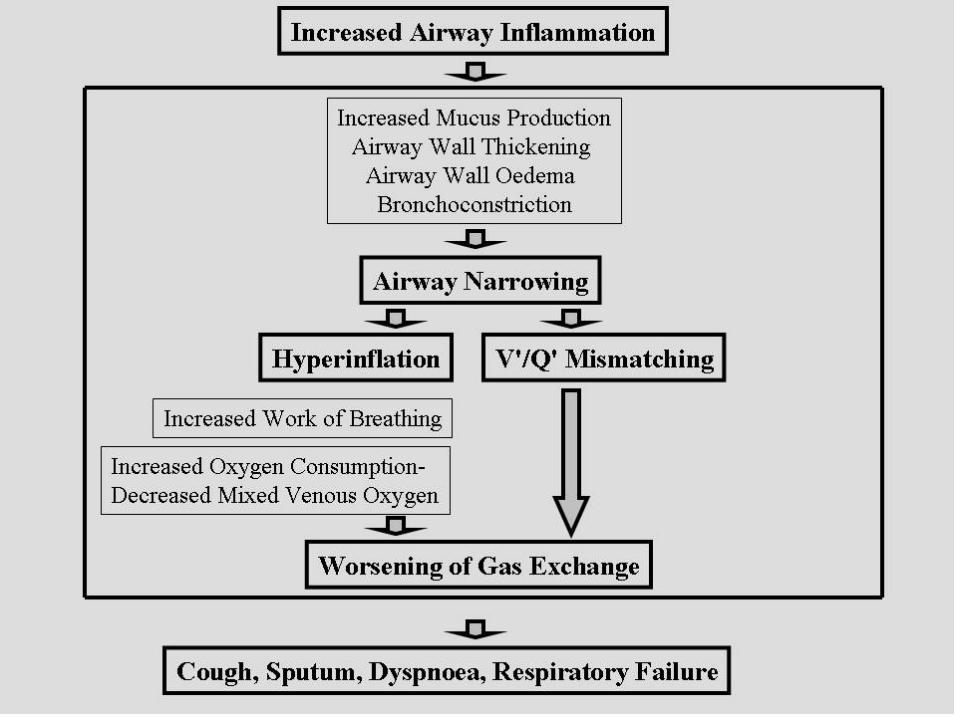
Schematic presentation of the main pathophysiologic events of COPD exacerbations, starting from increased airway inflammation.

A brief summary of the pathophysiologic events that may link airway inflammation to the symptoms of COPD exacerbations and to respiratory failure is given in figure 1. Airway narrowing, caused by increased inflammation, leads to expiratory flow limitation and dynamic hyperinflation. Dynamic hyperinflation in turn increases work of respiratory muscles and oxygen consumption, resulting in decreased mixed venous oxygen tension[91]. Airway narrowing also increases ventilation/perfusion inequality, because a greater proportion of blood flow is diverted through lung units with low V'/Q' ratios[91]. The combination of increased ventilation/perfusion inequality and decreased mixed venous oxygen tension significantly worsen gas exchange in patients with severe COPD exacerbations.

Increased airway inflammation would be also expected to increase lung tissue oxygen demand and oxygen consumption. In patients with acute lung injury no relation has been found between pulmonary oxygen consumption and lung inflammation, but there is no relevant data in COPD exacerbations[92].

### Anti-inflammatory therapy for exacerbations

Despite the increasing recognition of the importance of airway inflammation in the development of COPD exacerbations, there is still confusion regarding the role of anti-inflammatory strategies in the prevention and treatment of COPD exacerbations[93,94]. There are studies showing a beneficial effect of inhaled steroids in preventing COPD exacerbations and systemic steroids in treating acute exacerbations, however the overall benefits for COPD patients in these studies are generally modest [95-97]. Considering that inflammation in stable COPD is steroid resistant, this should not come as a great surprise[98]. Although the majority of COPD patients respond modestly to steroid therapy, there might be subgroup of patients, those with eosinophilic pattern of inflammation during exacerbations that may benefit the most from steroid administration. There is already evidence that stable patients with high sputum eosinophil counts are steroid responsive, but there is no relevant data on COPD exacerbations[99,100]. Identifying patients that may benefit from systemic steroid administration during exacerbations is of great importance, due to the serious adverse events frequently observed with this kind of treatment[97].

Other drugs with anti-inflammatory properties, like methylxanthines and mucolytic agents seem not to be effective on COPD exacerbations[8]. B-agonists are mainly administered as bronchodilators, although they may also have an anti-inflammatory role[101]. Novel drugs aimed at inhibiting targets, including NO synthase, phosphodiesterase 4, proteases and various inflammatory mediators have not been tested during exacerbations yet.

## Conclusion

It has been long postulated that airway inflammation may be increased during COPD exacerbations and this may be involved in the pathophysiology of exacerbations. There is now sufficient data to support such a hypothesis. Firstly, there are accumulating observations for increased inflammation during COPD exacerbations. Secondly, specific aetiological factors for this increase have been identified. Thirdly, possible mechanisms that may link airway inflammation with the pathophysiology of exacerbations have been unmasked. Despite significant advances in our understanding of the role of inflammation in COPD exacerbations, the existing anti-inflammatory treatments remain modest and there is little overall benefit for the patient. Further research is needed to target therapies to the appropriate patient populations and to develop new therapeutic strategies.

## Competing interests

The author(s) declare that they have no competing interests.
